# Primary and Secondary Mitochondrial Diseases: Etiologies and Therapeutic Strategies

**DOI:** 10.3390/jcm11144209

**Published:** 2022-07-20

**Authors:** Daniela Valenti, Rosa Anna Vacca

**Affiliations:** Institute of Biomembranes, Bioenergetics and Molecular Biotechnologies (IBIOM), National Research Council (CNR), Via G. Amendola122/O, 70126 Bari, Italy

Mitochondria are complex and multifaceted organelles that constitute a dynamic network of signaling platforms playing a pivotal role in cellular energy-generating processes. Optimal mitochondrial efficiency is fundamental for cellular life; it follows that mitochondrial alterations could be critically involved in the pathogenesis of several diseases, triggering the development a wide range of metabolic, neurodegenerative, immune, and neoplastic disorders [1].

Thousands of mitochondria can be present in a cell, the number of which depends on its function and metabolic activity. Mitochondria are the only cellular organelles containing their own DNA up to 10 copies of mitochondrial DNA (mtDNA) per mitochondrion. 

Repairing mutations in mtDNA occurs in a less efficient way than that of nuclear DNA (nDNA); therefore, the mutation rate of mtDNA is 10–20 times higher than that of nDNA [2]. Even within the same cell, some copies of mtDNA can have germline mutations, while other copies of mtDNA are wild-type, this one aspect characterizing the heteroplasmy. When the zygote divides, germline mutations in the mother’s mtDNA can end up in all cells, or only in specific cell lines, with other cell lines having a wild-type genetic karyotype [3,4,5].

Mitochondrial diseases (MDs) refer to a heterogeneous group of genetically transmitted multisystem disorders caused by impaired mitochondrial function due to mutations in mtDNA or nDNA [6]. Some affected genes encode proteins involved in mitochondrial transport proteins, enzymes, assembly factors, signaling proteins, pore proteins, and fusion/fission proteins of the dynamic mitochondrial network [7]. MDs can also be caused by mutations in genes with various functions or structural RNAs, such as transfer RNA (tRNA) and ribosomal RNA (rRNA), also including mutations in non-coding regions (e.g., mtDNA D-loops) [6]. The genes most frequently affected by mutations are those encoding the mitochondrial proteins of oxidative phosphorylation (OXPHOS) apparatus involved in the production of ATP, the major cellular energy charge [7]. MDs may manifest in any organ or tissue, but more frequently affect those with major energy requests [8]. MDs can manifest a clinical phenotype of a syndrome, defined as a collection of characteristics, frequently expressed as acronyms [9].

The terms primary and secondary mitochondrial diseases are used to describe mitochondrial pathophysiology. Primary mitochondrial diseases (PMD) are mitochondrial disorders caused by germline mutations in mtDNA and/or nDNA genes that encode either OXPHOS structural proteins or mitochondrial proteins of the complex machinery needed to carry out the OXPHOS process [3,7]. By distinguishing from PMD, secondary mitochondrial disorders (SMD) are elicited by mutations in other genes not related to OXPHOS. SMD can also be acquired by many other conditions, such as alterations in pathways regulating mitochondrial functions or adverse factors causing oxidative stress that can lead to secondary mitochondrial dysfunction and affect or worsen diseases, including neurodevelopmental and neurodegeneration diseases as well as diabetes, cancer, and aging [3,6]. SMD can be inherited or acquired differently from PMD, which can only be inherited; therefore, distinguishing whether mitochondrial dysfunction is inherited or acquired is an extremely critical and challenging point.

Diagnoses of PMD or SMS are based on one or more mitochondrial disease criteria (MDC) scoring systems designed to analyze mitochondrial energy-producing function, evaluating specific biochemical, clinical, and molecular characteristics and evaluating certain clinical or laboratory findings and classifying them into probable, possible, or unlikely diagnoses of mitochondrial diseases [4]. Although there is an important distinction between PMD and SMD, they can have overlapping symptoms and signs, making a correct diagnosis difficult to obtain. There are currently no consent guidelines commonly used for the diagnosis of mitochondrial diseases distinguishing whether they are primary or secondary due to the significant variable expressivity and incomplete penetrance. 

Contributions to this Special Issue may cover all research aspects related to advancements in the etiology, diagnosis, and therapeutic strategies for managing both primary and secondary mitochondrial disorders. We cordially invite scientists involved in basic research, as well as in preclinical and clinical studies, to submit their original research or review manuscripts to this Special Issue.
